# The efficacy of polyglycolic acid felt reinforcement in preventing postoperative pancreatic fistula after pancreaticojejunostomy in patients with main pancreatic duct less than 3 mm in diameter and soft pancreas undergoing pancreatoduodenectomy (PLANET-PJ trial): study protocol for a multicentre randomized phase III trial in Japan and Korea

**DOI:** 10.1186/s13063-019-3595-x

**Published:** 2019-08-09

**Authors:** Kazuto Shibuya, Jin-Young Jang, Sohei Satoi, Masayuki Sho, Suguru Yamada, Manabu Kawai, Hongbeom Kim, Song Cheol Kim, Jin Seok Heo, Yoo-Seok Yoon, Joon Seong Park, Ho Kyoung Hwang, Isaku Yoshioka, Toshio Shimokawa, Hiroki Yamaue, Tsutomu Fujii

**Affiliations:** 1 Department of Surgery and Science, Graduate School of Medicine and Pharmaceutical Sciences, University of Toyama, 2630 Sugitani, Toyama, 930-0194 Japan; 2 Department of Surgery, Cancer Research Institute, Seoul National University College of Medicine, Seoul, Republic of Korea; 3 Department of Surgery, Kansai Medical University, Hirakata, Japan; 4 Department of Surgery, Nara Medical University, Kashihara, Japan; 5 Department of Gastroenterological Surgery (Surgery II), Nagoya University Graduate School of Medicine, Nagoya, Japan; 6 Second Department of Surgery, Wakayama Medical University, Wakayama, Japan; 7 Department of Surgery, Asan Medical Centre, Seoul, Republic of Korea; 8 Department of Surgery, Samsung Medical Centre, Seoul, Republic of Korea; 9 Department of Surgery, Seoul National University Bundang Hospital, Seoul, Republic of Korea; 10 Department of Surgery, Gangnam Severance Hospital, Seoul, Republic of Korea; 11 Department of Surgery, Severance Hospital, Seoul, Republic of Korea; 12 Clinical Study Support Centre, Wakayama Medical University, Wakayama, Japan

**Keywords:** Pancreatoduodenectomy, Pancreaticojejunostomy, Postoperative pancreatic fistula, Polyglycolic acid felt, International multicentre study

## Abstract

**Background:**

Partial pancreatoduodenectomy is performed for malignant and benign diseases of the pancreatic head region. The procedure is considered highly difficult and highly invasive. Postoperative pancreatic fistula (POPF) is an important complication because of several consequent complications, including intraabdominal haemorrhage, often increasing hospital stays and surgical mortality. Although many kinds of pancreaticojejunostomy aimed at reducing POPF have been examined to date, the technique has not yet been standardized. We devised a new method using double-coated polyglycolic acid felt after pancreaticojejunostomy. The aim of the PLANET-PJ trial is to evaluate the superiority of polyglycolic acid felt reinforcement in preventing POPF after pancreaticojejunostomy in patients undergoing partial pancreatoduodenectomy to previous anastomosis methods.

**Methods:**

Patients diagnosed with pancreatic or periampullary lesions in whom it is judged that the main pancreatic duct diameter was 3 mm or less on the left side of the portal vein without pancreatic parenchymal atrophy due to obstructive pancreatitis are considered eligible for inclusion. This study is designed as a multicentre randomized phase III trial in Japan and the Republic of Korea. Eligible patients will be centrally randomized to either group A (polyglycolic acid felt reinforcement) or group B (control). In total, 514 patients will be randomized in 31 high-volume centres in Japan and Republic of Korea. The primary endpoint is the incidence of POPF (International Study Group of Pancreatic Surgery grade B/C).

**Discussion:**

The PLANET-PJ trial evaluates the efficacy of a new method using double-coated polyglycolic acid felt reinforcement for preventing POPF after pancreaticojejunostomy. This new method may reduce POPF.

**Trial registration:**

ClinicalTrials.gov, NCT03331718. University Hospital Medical Information Network Clinical Trials Registry, UMIN000029647. Registered on 30 November 2017. https://upload.umin.ac.jp/cgi-open-bin/ctr/ctr_view.cgi?recptno=R000033874

**Electronic supplementary material:**

The online version of this article (10.1186/s13063-019-3595-x) contains supplementary material, which is available to authorized users.

## Background

Partial pancreatoduodenectomy is performed on malignant and benign diseases of the pancreatic head region. The procedure is considered highly difficult and highly invasive. Pancreaticojejunostomy requires a high level of surgical technique for anastomosing the residual pancreas and jejunum. When implementing partial pancreatoduodenectomy, a high-volume centre with highly skilled surgeons is recommended. The incidence of complications after partial pancreatoduodenectomy is reported to be 30–65%, higher than those of other digestive tract surgeries [1–10]. Among these complications, postoperative pancreatic fistula (POPF) is generally recognized as an important complication, because intraabdominal haemorrhage can be fatal. Increases in postoperative hospital stay and surgical mortality rates are also problems.

POPF was first standardized and reported in 2005 [11]. According to the latest International Study Group of Pancreatic Surgery (ISGPS) criteria reported in 2016 [12], the former “grade A POPF” is now redefined and called a “biochemical leak” because it has no clinical importance and is no longer referred to a true pancreatic fistula. Therefore, POPF has only grade B and grade C. Grade B requires a change in the postoperative management; drains are either left in place for > 3 weeks or repositioned through endoscopic or percutaneous procedures. Grade C refers to those POPFs that require reoperation or lead to single or multiple organ failure and/or mortality attributable to the pancreatic fistula.

Pancreaticojejunostomy generally involves a combination of suture between the pancreatic parenchyma and the seromuscular layer of the jejunum, and duct-to-mucosa suture. A clinical study regarding the various kinds of pancreaticojejunostomy was reported for the purpose of lowering the frequency of POPF; nevertheless, the frequency of more than grade B POPF remains approximately 10–20% [13–15]. In soft pancreas patients with unexpanded pancreatic ducts, the risk is further elevated to over 30% [16].

Polyglycolic acid (PGA) felt is a felt-like absorbable suture-reinforcing material. It is generally used to reinforce sutures of fragile tissues such as the lung, bronchi, liver and gastrointestinal tract and to reinforce a wide range of tissue defects. Regarding pancreaticojejunostomy using a PGA felt, the incidence of POPF formation was lower in some retrospective studies [17, 18]; on the other hand, no significant difference was found other studies [19, 20]. As already described, pancreaticojejunostomy with reinforcement of a PGA felt aimed at reducing POPF has yet to be fully examined.

We reported significant decreases of POPF thanks to the ingenuity of pancreaticojejunostomy in a single-institution matched historical-control study [21]. In distal pancreatectomy, we have verified the effect of oral food intake on POPF [22], and are participating in a multicentre prospective randomized trial associated with POPF [23].

Here, we have devised a new method using double-coated PGA felt for pancreaticojejunostomy in patients with a small diameter of the main pancreatic duct. This study is a multicentre randomized phase III trial in Japan and Republic of Korea to verify the usefulness of this double coating of PGA felt preventing POPF.

## Methods/design

### Aim

The aim of the PLANET-PJ trial is to evaluate the efficacy of PGA felt reinforcement in preventing POPF after pancreaticojejunostomy in patients with a main pancreatic duct less than 3 mm in diameter and a soft pancreas undergoing partial pancreatoduodenectomy.

### Study population

Patients undergoing pancreaticojejunostomy without a dilated main pancreatic duct (MPD) (so-called soft pancreas) and parenchymal atrophy or pancreatitis are eligible. There are no restrictions to the type of disease. Detailed eligibility criteria are presented in Table 1.

**Table 1:** Eligibility criteria

Inclusion criteria	
(1) Pancreatic or periampullary lesions requiring pancreatoduodenectomy	
(2) Planned pancreaticojejunostomy including duct-to-mucosa anastomosis	
(3) Main pancreatic duct diameter ≤ 3 mm on the left side of the portal vein on preoperative imaging (computed tomography or magnetic resonance imaging)	
(4) Performance status (Eastern Cooperative Oncology Group scale): 0–1 at the time of enrolment	
(5) Age 20 years or older	
(6) Adequate organ function	
(a) Leukocyte count ≥ 2500 mm^3^, ≤ 14000 mm^3^	
(b) Haemoglobin ≥ 9.0 g/dl	
(c) Platelet count ≥ 100,000 mm^3^	
(d) Total bilirubin ≤ 2.0 mg/dl (does not apply to patients with obstructive jaundice)	
(e) Creatinine ≤ 2.0 mg/dl	
(7) Ability to understand and willingness to sign written informed consent document	
Exclusion criteria	
(1) Planned pancreatogastrostomy	
(2) Laparoscopic or laparoscope-assisted pancreatoduodenectomy	
(3) Pancreatic parenchymal atrophy or calcification due to chronic pancreatitis	
(4) Neoadjuvant treatment including chemotherapy or radiotherapy	
(5) History of upper abdominal surgery (both of open and laparoscopic) except cholecystectomy	
(6) Emergency surgery	
(7) Arterial reconstruction such as superior mesenteric artery, common hepatic artery or celiac artery	
(8) Severe ischaemic heart disease	
(9) Severe liver dysfunction due to liver cirrhosis or active hepatitis	
(10) Severe respiratory disorder requiring oxygen	
(11) Chronic renal failure with dialysis	
(12) Requiring resection of other organs (liver or colon) during pancreatoduodenectomy	
(13) Immunosuppressive treatment	
(14) History of severe hypersensitivity to polyglycolic acid felt or fibrin glue	
(15) Other severe drug allergies	
(16) Contrast media allergy for both iodine and gadolinium	
(17) Active duplicate cancer thought to affect adverse events	
(18) Severe psychological or neurological disease	
(19) Drug or alcohol abuse	
(20) Planned use of octreotide	

### Study design

This study is designed as a multicentre randomized phase III trial in Japan and Republic of Korea. A total of 514 patients will be included, and 31 leading institutions and hospitals in Japan and Republic of Korea will participate in the PLANET-PJ trial (Additional file 1). The registration period is scheduled for 3 years, and the follow-up period will be 3 months.

### Randomization

**Fig. 1:**
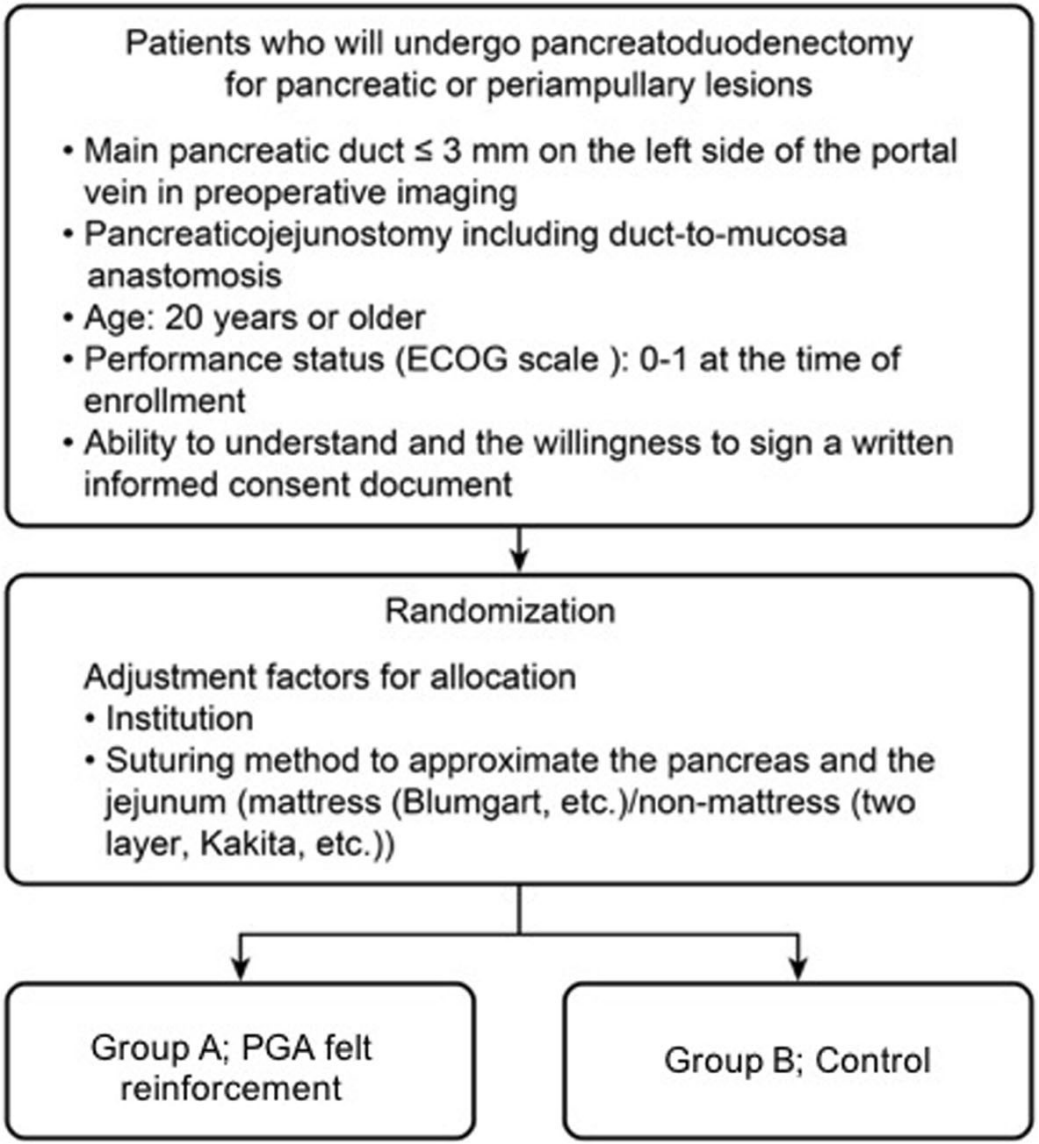
Flow diagram of the PLANET-PJ trial. ECOG Eastern Cooperative Oncology Group, PGA polyglycolic acid

After confirmation of eligibility, including written informed consent, patients are randomized in a 1:1 allocation ratio to either group A (polyglycolic acid felt reinforcement) or group B (control), preoperatively. Central randomization and registration will be applied, using the Electronic Data Capture (EDC) system (Seoul National University, Medical Research Collaborating Center). After being assessed for eligibility at registration, patients will be centrally randomized to either group A or group B. To minimize background bias between the two groups, this study is stratified for institution and suturing method to approximate the pancreas and the jejunum (mattress or non-mattress). We use Pocock and Simon’s minimization method for random assignment and the Mersenne Twister for random number generation. See Fig. 1 for a flow diagram of the PLANET-PJ trial.

All patients are blinded to the surgical approach that they will be receiving, and they are required to sign an informed consent before enrolling in this study [24]. Blinding of the surgeons is not possible, owing to the different techniques used during the operation. However, the result assessment will be made by an independent researcher (TS) who will be blinded to the surgical procedures. Physicians, study nurses and statisticians are blinded to the surgical approach as well.

### Endpoints

This clinical trial will primarily evaluate the incidence of POPF. The secondary endpoints will be the length of drain placement after surgery, length of the hospital stay after surgery, incidence of overall biochemical leak and POPF, incidence of POPF by each suturing method to approximate the pancreas and the jejunum, incidence of delayed gastric emptying (DGE), incidence of intraabdominal abscess, incidence of post-pancreatectomy haemorrhage (PPH), incidence of interventional drainage, incidence of overall postoperative complications, incidence of POPF-related complications (POPF + DGE + abscess + PPH), incidence of 3-month mortality and incidence of reoperation.

Biochemical leak, POPF [12], DGE [25] and PPH [26] are defined and graded according to the International Study Group of Pancreatic Surgery (ISGPS) criteria and Clavien–Dindo classification [27]. Postoperative complications other than POPF, DGE and PPH are graded according to Clavien–Dindo classification [27]. Then, the comprehensive complication index was evaluated as a secondary outcome [28].

Subgroup analyses will be planned per anastomosis or pancreatic stent. These secondary outcomes will be exploratory.

### Sample size estimation

This trial was designed to evaluate the efficacy of group A compared with group B in terms of the incidence of POPF. In cases using PGA felt at the pancreaticojejunostomy in partial pancreatoduodenectomy with an MPD diameter of 3 mm or less, the incidence of POPF was 15.5% [18, 19]. The incidence of POPF was 26% in a previous report without PGA felt at the pancreaticojejunostomy in partial pancreatoduodenectomy with an MPD diameter of 3 mm or less [29]. When statistical analysis is performed for a significance level of α = 0.05 (two-sided) in a superiority design, 231 patients are calculated to be required per arm, with a power 100(1 – β) of more than 80%, under the assumption that a small number of patients may be deemed ineligible and may thus be excluded from the analysis. Furthermore, as approximately 10% of the patients are expected to be ineligible for surgery, the sample size was eventually increased to 514 patients (257 patients per arm). This sample size was calculated using software PASS 15.0.6.

In the evaluation of the secondary endpoints, a hypothesis test will be used for comparison between both groups, although formal power calculations for these analyses have not been performed.

### Statistical analysis plan

All statistical analyses will be performed using the full analysis set (FAS) under the intention-to-treat principle. The significance level of this test is 5% (two-sided) and the confidence coefficient is 0.95. The Wilcoxon rank-sum test will be used for comparison of continuous variables. The Fisher’s exact test will be used for comparison of categorical variables. Kaplan–Meier curves will be used to represent time-to-event variables, and hazard ratios and 95% confidence intervals will be estimated by the Cox proportional hazard model. *P* < 0.05 will be considered significant.

### Interventions

#### Surgical procedure

In both groups, the subjects are patients without dilated MPD (so-called soft pancreas) and parenchymal atrophy or pancreatitis. There are no regulations regarding the type of disease, the degree of lymph node dissection, portal vein resection or the type of suture used. Patients undergoing pancreaticojejunostomy with duct-to-mucosa anastomosis will be eligible for inclusion; however, there are no regulations regarding the suturing method to approximate the pancreas and the jejunum (Kakita method, two-layer suturing method, modified Blumgart method, etc.). Pancreaticogastrostomy and the invagination method are not permitted.

The MPD diameter will be measured before pancreaticojejunostomy to reconfirm that it is 3 mm or less. If it exceeds 3 mm, the protocol treatment will be terminated. There are no restrictions to the placement of pancreatic stent (yes/no) and the method (external/internal). The presence or absence of a pancreatic stent is registered postoperatively as intraoperative information in the EDC system. Even if the policy of the suturing method to approximate the pancreas and the jejunum is changed during surgery (mattress or non-mattress), the study treatment shall be continued. The anastomotic drains will be placed in all enrolled patients. There are no restrictions to the type and number of anastomotic drains; however, a closed-type drain must be used and must be placed around the pancreaticojejunostomy.

**Fig. 2:**
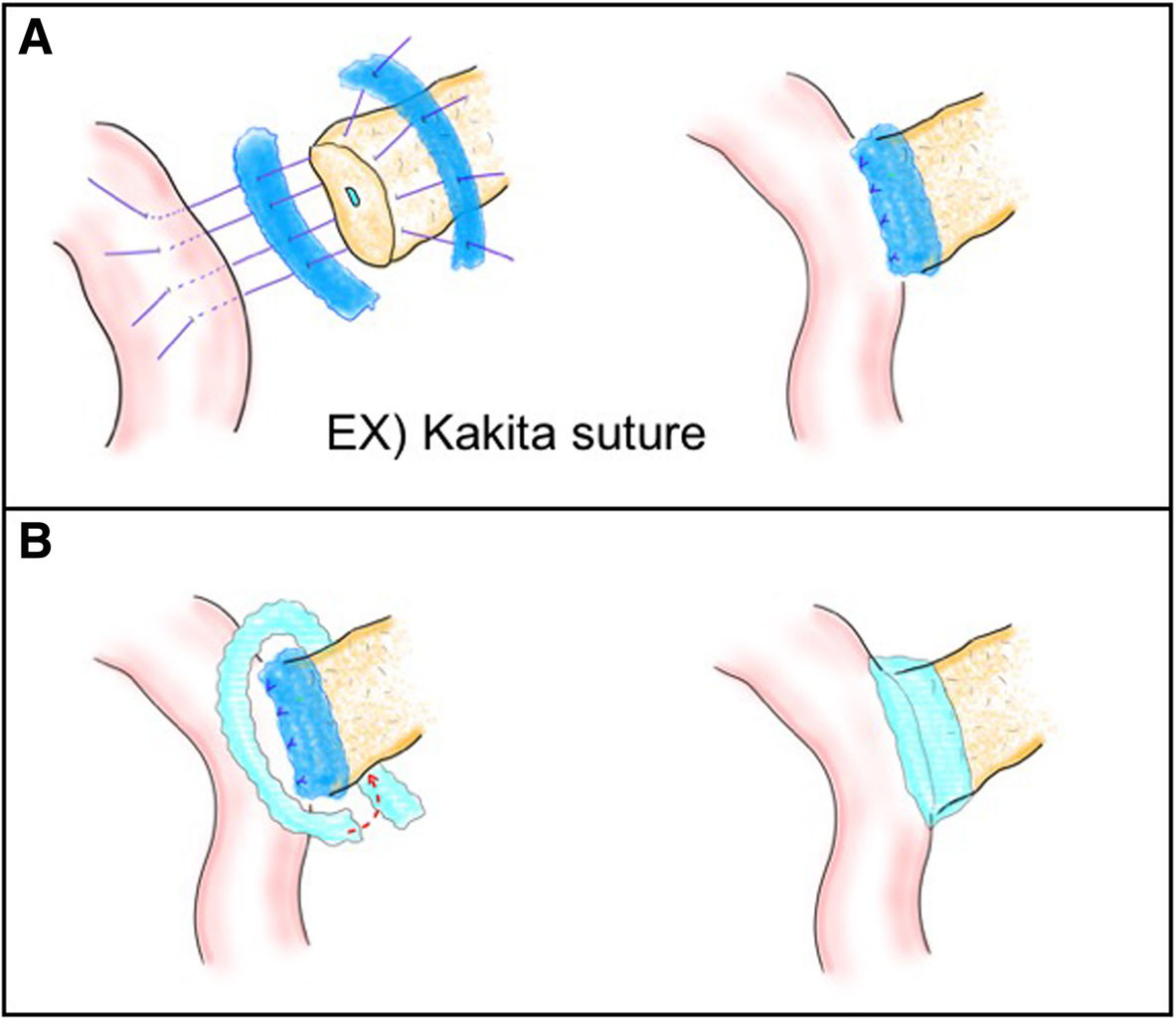
Method using double-coated polyglycolic acid (PGA) felt during pancreaticojejunostomy. **a** A 0.3-mm thick PGA felt (Neoveil®; GUNZE, Japan) will be pasted on the ventral side and the dorsal side of the pancreatic parenchyma. **b** Before abdominal closure, a 0.15-mm thick PGA felt will be further covered around the anastomotic site and fibrin glue will be sprayed

In group B, pancreaticojejunostomy will be performed as already described. In group A, during pancreaticojejunostomy: a 0.3-mm thick PGA felt (Neoveil®; GUNZE, Japan) will be pasted on the ventral side and the dorsal side of pancreatic parenchyma, through which suture between the pancreatic parenchyma and the jejunum will be performed (Fig. 2a); and before abdominal closure (after completion of all reconstruction, after washing in the abdominal cavity), a 0.15-mm-thick PGA felt will be further covered around the anastomotic site and fibrin glue will be sprayed (Fig. 2b).

#### Intraoperative photography

To confirm that the surgical procedure is performed correctly by central judgement, intraoperative photographs of the surgical fields will be needed in both groups. The anastomotic site of pancreaticojejunostomy will be photographed with a digital camera immediately after pancreaticojejunostomy (both in groups A and B) and after spraying with fibrin glue (only in group A). Photographs will be sent by email to the administrative office (University of Toyama) within 4 weeks after surgery. Central judgement will be conducted every 6 months for all of the registered patients by the members of the central review committee. If the procedure is not appropriate, the case will be excluded. The central judgement committee has respective members in charge of group A or group B. They will judge to exclude the inappropriate cases from a per-protocol analysis set, and we will review the number of excluded cases in the final report/publication.

#### Postoperative management

Blood/ biochemical examination and amylase level measurements of anastomotic drains will be performed on postoperative day (POD) 1 and POD 3. If the amylase level of anastomotic drains on POD 1 is 5000 IU/L or less, we recommend drain removal on POD 4–6. In patients with POPF or infectious signs that require therapeutic drainage, drainage should be continued. Prior to drain removal, a CT/MRI check must be performed to confirm the intra-abdominal situation. After POD 4, blood/biochemical examination or drain amylase-level measurements just before removing the anastomotic drains are performed according to the physician’s discretion. Use of a prophylactic antimicrobial agent after surgery or prophylactic routine exchange of anastomotic drains shall not affect the definition of POPF to be classified. In the case of continued drainage after POD 4–6, we recommend drain removal when either or both of the following conditions continue for 2 consecutive days: the amylase level in the drainage fluid is less than three times the upper limit of institutional normal serum amylase level; or the amount of the drainage fluid is 20 mL/day or less.

#### Postoperative follow-up

**Fig. 3:**
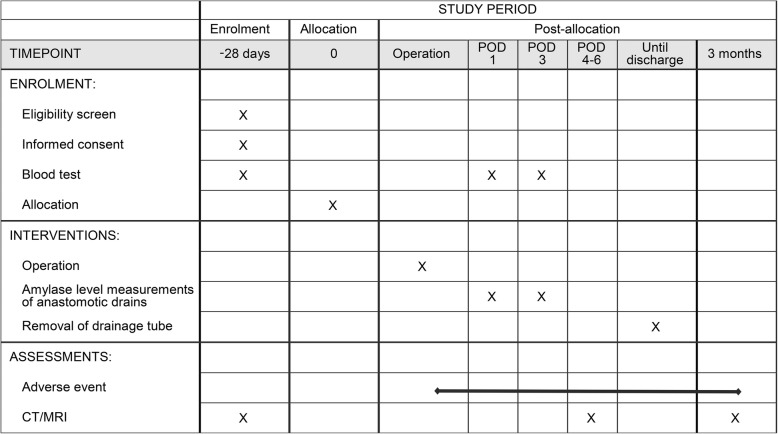
Study calendar. CT/MRI computed tomography/magnetic resonance imaging, POD postoperative day

Follow-up 3 months after surgery to observe the occurrence of postoperative complications, re-hospitalization or re-operation will be a part of the protocol treatment. Three months after surgery, contrast-enhanced CT (or MRI) will be taken to measure the MPD diameter in both groups. See Fig. 3 for the study calendar.

#### Interim analysis

Interim analysis will be performed once, taking multiplicity into account using the Lan–DeMets method with O’Brien and Fleming type boundaries. The monitoring committee will independently review the interim analysis report and stop the trial early if necessary.

#### Monitoring

Central monitoring will be performed once a year by an independent data monitoring committee. The monitoring committee will collect information on the status of accumulation, inclusion/exclusion criteria, serious adverse events, etc., and strive to provide feedback to participating institutions for early resolution if there are any problems. The monitoring committee will also report the serious adverse events to the committee of efficacy and safety assessment. The PLANET-PJ trial will be conducted in Japan and Republic of Korea; therefore, monitoring committees will be established in each country.

#### Efficacy and safety assessment

If a serious adverse event occurs after the start of the study, or a problem that requires early termination of the entire study occurs, the committee of efficacy and safety assessment will deliberate with the principal investigator. The committee of efficacy and safety assessment will also be set up in both countries and will discuss the results of monitoring and adverse events during the trial.

## Discussion

Partial pancreatoduodenectomy is an advanced procedure requiring a high level of surgical technique. With the development of surgical techniques and perioperative management in recent years, surgical-related mortality rates have become less than 5%. Nevertheless, the incidence of complications remains high at 30–65% [1–10]. In particular, the high frequency of POPF is problematic, because the various complications according to POPF, including intraabdominal haemorrhage or intraabdominal abscess, can be fatal.

There is no evidence to prove a safe and reliable procedure of pancreaticojejunostomy. We planned this study to evaluate the efficacy of a new method using double-coated PGA reinforcement to prevent POPF after pancreaticojejunostomy. This trial is a multicentre randomized phase III trial in Japan and Republic of Korea that is the first international study in pancreatic surgery. This study is designed to evaluate the usefulness of group A (double coating of PGA felt) compared with group B (without PGA felt) in terms of the frequency of POPF as the primary endpoint. Upon randomization, the eligible subjects will be stratified by the institution and suturing method to approximate the pancreas and the jejunum (mattress or non-mattress). The reason is that these factors may affect the incidence of POPF. Therefore, we will set these two factors for allocation in this study for appropriate analysis and evaluation. Moreover, we randomize the patients preoperatively after evaluating appropriateness in the CT image just before surgery. It would be ideal to randomize during surgery after the measurement of the diameter of the main pancreatic duct, but this is very difficult because of the situation of the participating facilities in this study.

All patients are blinded to the surgical approach that they will be receiving, and they are required to sign an informed consent before enrolling in this study [24]. However, blinding of the surgeons is not possible, owing to the different techniques used during the operation. This can be a potential source of inevitable bias. The anastomotic drains will be placed in all enrolled patients because measurement of the amylase level in the anastomotic drains is required for assessment of POPF, although recent meta-analysis revealed that pancreatic resection with or without abdominal drainage results in similar rates of mortality, morbidity and reintervention [30].

Industry-funded RCTs are prone to report positive results. This study is funded by GUNZE LIMITED. Probst et al. [31] reported that industry involvement in surgical research has to ensure scientific integrity and independence, and has to be based on full transparency. In this study, employees of GUNZE LIMITED did not have access to the data during the trial, did not participate in the data analysis and did not participate in the preparation of the manuscript other than to review it. The design and implementation of this study is being conducted only by the investigators (TF, KS, J-YJ, MK, MS, SS, SY, HY, HK, SCK, JSH, Y-SY, JSP, HKH). GUNZE LIMITED has nothing to do with this study other than funding.

If the usefulness of this new double coating of PGA felt method is proven, we believe that this trial may provide evidence for lowering the incidence of POPF, to the great benefit of patients who undergo partial pancreatoduodenectomy and of the medical economy. Furthermore, in patients with malignant disease, postoperative adjuvant chemotherapy might be introduced promptly, to lead to prolongation of overall survival (Additional file 2).

## Trial status

The PLANET-PJ trial was opened in October 2018. At the time of submission of this article (November 2018), the protocol is version 1.1. The completion date is estimated to be June 2021.

## Additional files

Additional file 1: Institution list (DOCX 15 kb)

Additional file 2: SPIRIT 2013 Checklist: Recommended items to address in a clinical trial protocol and related documents (DOC 123 kb)

## Data Availability

The datasets used and/or analysed during the current study are available from the corresponding author on reasonable request.

## Abbreviations

DGE: Delayed gastric emptying
FAS: Full analysis set
ISGPS: International Study Group of Pancreatic Surgery
MPD: Main pancreatic duct
PGA: Polyglycolic acid
POPF: Postoperative pancreatic fistula
PPH: Post-pancreatectomy haemorrhage
