# Supplementary material for: The efficacy of polyglycolic acid felt reinforcement in preventing postoperative pancreatic fistula after pancreaticojejunostomy in patients with main pancreatic duct less than 3 mm in diameter and soft pancreas undergoing pancreatoduodenectomy (PLANET-PJ trial): study protocol for a multicentre randomized phase III trial in Japan and Korea
Source: Trials. 2019 Aug 9;20:490. doi: 10.1186/s13063-019-3595-x (PMC6688253; doi:10.1186/s13063-019-3595-x)
Supplement: Supplementary file 1 — Institution list (DOCX 15 kb) [file 13063_2019_3595_MOESM1_ESM.docx]

Additional file 1. Institution list

| Participating institutions | |
| --- | --- |
| Japan | Korea |
| Kansai Medical University | Seoul National University Hospital |
| Kumamoto University | Seoul National University Bundang Hospital |
| Nagoya University | Asan Medical Center |
| Nara Medical University | Samsung Medical Center |
| Osaka City University | Severance Hospital |
| Osaka University | Gangnam Severance Hospital |
| Shiga Medical University | Korea University Guro Hospital |
| Shimane University | Ewha Womans University |
| Tokyo Medical and Dental University | Boramae Hospital |
| Tokyo Medical University | Chung Ang University Hospital |
| Wakayama Medical University | Chonbuk National University Hospital |
| University of Toyama | Soonchunhynag Bucheon Hospital |
|  | Busan National University Hospital |
|  | Yeouido St. Mary's Hospital |
|  | Kuyng Hee University Hospital |
|  | Gyeongsang National University Hospital |
|  | Chung Nam National University Hospital |
|  | Bucheon St. Mary's Hospital |
|  | Ulsan University Hospital |
